# Increased nuclear translation of YAP might act as a potential therapeutic target for NF1-related plexiform neurofibroma

**DOI:** 10.7150/ijms.52431

**Published:** 2021-03-03

**Authors:** Jia-Liang Liu, Yuan-He You, Zhuo-Wei Tian, Meng Xiao, Jia-Wei Zheng, Yan-An Wang, Zhong Du

**Affiliations:** 1 Department of Oral and Maxillofacial-Head and Neck Oncology, Shanghai Ninth People's Hospital, Shanghai Jiao Tong University, School of Medicine, Shanghai 200011, P.R. China.; 2 National Clinical Research Center for Oral Diseases, Shanghai 200011, P.R. China.; 3 Shanghai Key Laboratory of Stomatology & Shanghai Research Institute of Stomatology, Shanghai 200011, P.R. China.

**Keywords:** Plexiform neurofibroma, head and neck, NF1, YAP, Selumetinib.

## Abstract

Plexiform neurofibroma (pNF) in the head and neck is a characteristic feature in patients with neurofibromatosis type 1 (NF1) and is associated with significant disfigurement and psychological distress. Yes-associated protein (YAP), the key molecule involved in the Hippo pathway, is a vital transductor that regulates the proliferation and remyelinating of Schwann cells. The functional status of YAP and its feasibility as a potential target are still unknown in pNF. A total of 17 pNF tumor tissue specimens from the head and neck were collected at the Ninth People's Hospital, Shanghai Jiao Tong University School of Medicine. Histologically, diagnosis of the Schwann cell region in pNF was achieved with hematoxylin-eosin staining, positive reactions for S100, SOX10, ERK and p-ERK, and low identification of Ki67 and SMA. Compared with normal nerve tissue, obviously increased nuclear YAP was detected in the Schwann cell region of pNF, with a mean nuclear staining rate of 67.11%. Based on the *shNF1* Schwann cell model (the RSC96 cell line), with upregulated expression of RAS, ERK and p-ERK, p-YAP (Ser127) and p-YAP (Ser397) were significantly decreased and total YAP and nuclear YAP were increased. According to a confocal assay, the interference of *shNF1* substantially promoted YAP nuclear translocation. Compared with control Schwann cells, the YAP inhibitor CA3 might have a more sensitive effect (IC_50_: NC=0.96±0.04, shNF1=0.71±0.02, *P*<0.05) on the *shNF1* Schwann cell model than the classic MEK1/2 inhibitor selumetinib (IC_50_: NC=14.36±0.95, shNF1=24.83±0.98, *P*>0.05). For *in vivo* inhibition, the CA3 group and the selumetinib group displayed a similar inhibition effect with no significant difference. Increased nuclear translation and the functional state of YAP implies that the YAP-Hippo pathway might play an important role in the formation and remyelination of pNF. Compared with selumetinib, the YAP inhibitor can exhibit a similar but more sensitive effect on *NF1^-/-^* Schwann cells. These observations imply that YAP as a novel or adjuvant therapy target in the treatment of pNF.

## Introduction

Neurofibromatosis type 1 (NF1) is a frequent autosomal dominant hereditary tumor syndrome with a wide clinicopathologic spectrum. This disorder is characterized by café-au-lait maculae (CALM), skinfold freckling, iris Lisch nodules, and benign peripheral nerve sheath tumors (BPNSTs, neurofibromas). Neurofibroma can commonly be further classified into two main types, cutaneous neurofibroma (cNF) and plexiform neurofibroma (pNF). For these two subtypes, slowly growing pNF is counted as a characteristic clinical feature and is associated with significant disfigurement and psychological distress. Moreover, in approximately 10% of NF1 patients, pNF might undergo transformation to malignant peripheral nerve sheath tumors (MPNSTs), which are highly aggressive 1-3. Unfortunately, under the pressure of and uncontrollable intraoperative hemorrhage and other postoperative complications, surgical excision management still cannot stop the high reoccurrence or growth tendency.

Commonly, mutations in *NF1*, a tumor suppressor gene, are identified or inherited in NF1 patients. *NF1* encodes the protein neurofibromin, which functions as a suppressor of the RAS pathway. The loss of function of neurofibromin can cause hyperactivation of the RAS-RAF-MEK-ERK signaling pathway. Histologically, pNF is commonly composed of a mixture of proliferating Schwann cells, fibroblasts, mast cells, pericytes, endothelial cells, and inflammatory cells 2, 3. Compared with the other types of cells, heterozygous loss of *NF1* in the Schwann cell lineage is completely sufficient to induce pNF 4. Different genetically engineered mice have been used to identify the driver position of *NF1* derived from Schwann cells as well as *Nf1^flox/flox^;PostnCre^+^* mice 5, which established the pathogenic role of Schwann cells in pNF.

According to the latest clinical trials and considering inoperative situations, target inhibitors have been gradually considered as potential therapies. With acceptable response rates in phase I and II trials varying between 42% and 74%, the selective inhibitor for MEK1/2 selumetinib (AZD6244, ARRY-142886) was accepted as a New Drug Application by the United States Food and Drug Administration (FDA) for children with symptomatic pNF, representing a promising therapeutic option for NF1-related inoperable pNF 6-8. However, with its application in additional clinical trials, the side reactions of selumetinib have received more attention. The most adverse cumulative toxicities include nausea, vomiting, abdominal pain, diarrhea, oral mucositis, rash, dry skin, and/or elevated creatine phosphokinase levels, and each of which occurred in >50% of patients. No direct relationship was observed between neurofibroma volume and response, even at baseline, and a few patients might suffer from an increasing neurofibroma volume. Limited to test cycles, the long-term tolerability and duration of selumetinib treatment required to sustain clinical benefit are unknown. The notably slow shrinkage of pNF cannot offer immediate symptomatic relief 9. Thus, attempts to explore novel targets are still needed to develop or repurpose compounds for the treatment of pNF.

According to research related to neuroblastomas with hyperactivated MAPK signaling, increased transcriptional activity of yes-associated protein (YAP) in Schwann cells is a vital mechanism of MEK1/2 inhibition resistance 10, which implies a potential novel target for NF1. The Hippo-Lats-Yap pathway was also verified to act as a modifier of plexiform neurofibroma 11. YAP, the key molecule involved in the Hippo pathway, is a vital transductor between the cytoplasm and nucleus. As a downstream molecule, YAP dephosphorylation allows its translocation into the nucleus and its interaction with the TEAD family and other transcription factors to initiate transcription of multiple gene targets, which might stimulate cell proliferation, survival, phenotypic plasticity, therapy resistance, metastasis, extracellular matrix (ECM) alteration, tissue regeneration and repair, and inflammation 10, 12, 13. Mutations in the Hippo pathway appeared to play the vital role of acquired somatic mutations in neurofibroma growth behavior 14.

Based on these observations, we aimed to certify the underlying role of YAP in pNF. First, we detected significantly increased nuclear translation of YAP in NF1 patients. We subsequently established the *NF1^-/-^* Schwann cellular model via the *shRNA* approach and identified significantly increased nuclear translation and downregulated phosphorylation of YAP. Compared with selumetinib, via an *in vitro* or null murine assay, the YAP inhibitor CA3 displayed a similar potent inhibition effect with a lower dose. All of these observations suggest that YAP is a potential novel target for the treatment of NF1.

## Methods

### Patients and tissue samples

In total, 17 pNF tumor tissue specimens were collected from January 2009 to December 2019 at the Ninth People's Hospital, Shanghai Jiao Tong University School of Medicine (Shanghai, China). Informed consent was provided by all patients. All patients were diagnosed with pNF related to NF1 according to the diagnostic criteria approved by the National Institutes of Health (suffering from two or more of the following: six or more café-au-lait spots, two or more neurofibromas of any type, one or more plexiform neurofibromas, two or more Lisch nodules, a first-degree relative with NF1, etc.) 2, 3. The medical records of all patients were reviewed, and pathological classifications were carefully analyzed. This study was approved by the Ethics Committee of Shanghai Ninth People's Hospital.

### Histology and immunohistochemistry

All tissue was fixed, dehydrated, embedded in paraffin, sectioned at 3 μm, and stained with hematoxylin and eosin according to standard protocols. For immunohistochemistry staining, primary antibody incubations with anti-S100 (1:1000, ab183979, Abcam, USA), anti-YAP (1:100, ab52771, Abcam, USA), anti-SOX10 (1:100, ab227680, Abcam, USA), anti-Ki67 (1:1000, 27309-1-AP, Proteintech, USA), anti-SMA (1:100, 55135-1-AP, Proteintech, USA) antibodies, anti-ERK (1:100, 4695, CST, USA) and anti-pERK (1:100, ab201015, Abcam, USA) were performed. The sections were incubated with horseradish peroxidase-labeled secondary antibodies (GK500705, Gene Tech, China). The antibody signals were visualized using diaminobenzidine, and the sections were counterstained with hematoxylin. Microscopic examination of YAP immunohistochemical staining was conducted on the tissue samples by two blinded pathologists. The average intensity of YAP immunoreactivity was scored via 4 random sites located in the S100 positive area.

### Cell culture and transfection

The RSC96 cell line was obtained from the Cell Bank, Chinese Academy of Sciences. The cells were routinely cultured in Dulbecco's modified Eagle medium (DMEM) (12800017, Gibco, NY). DMEM was supplemented with 10% fetal bovine serum and 1% penicillin/streptomycin. All cells were cultured at 37°C and 5% CO_2_ in an incubator. Small hairpin RNA (*shRNA*), plasmids, and lentiviruses were synthesized by RiboBio Inc. (China). Cell transfection was performed using the Lipofectamine® 3000 Transfection Kit (L3000015, Invitrogen, USA).

### Western blot assay

Protein extraction and Western blot assays were conducted as previously described. Total protein was extracted with RIPA lysis buffer in the presence of a protein phosphatase inhibitor cocktail (78440, Pierce, USA). Following separation on 10% SDS polyacrylamide gels, the proteins were transferred onto polyvinylidene fluoride membranes and subsequently blocked in 5% skimmed milk for 1 hour at room temperature. The membranes were incubated with primary antibodies against NF1 (1:1000, 14623, Cell Signaling Technology, USA), YAP (1:1000, 14074, Cell Signaling Technology, USA), RAS (1:1000, 3339, Cell Signaling Technology, USA), ERK (1:1000, 4370, Cell Signaling Technology, USA), p-ERK (1:1000, 9101, Cell Signaling Technology, USA), and GAPDH (1:1000, 5174, Cell Signaling Technology, USA) overnight at 4℃. After three thorough washes, the membranes were incubated with horseradish peroxidase-linked secondary antibodies (GK500705, Gene Tech, China) at room temperature for 1 hour. Finally, the membranes were washed three times and visualized using an ECL kit (35050, Thermo Fisher, USA).

### Cell proliferation assay

To determine the optimal concentration of CA3 (S8661, Selleck Chemicals, USA) for the following studies, various concentrations of the YAP inhibitor (0.5, 0.6, 0.7, 0.8, 0.9, 1.0, 1.5, and 2.0 μmol/L) were used. To determine the optimal concentration of selumetinib (S1008, Selleck Chemicals, USA) for the following studies, various concentrations (0.05, 0.14, 0.41, 1.23, 3.7, 11.11, 33.33, 100, and 300 μmol/L) were used. A parallel set of 3,000 cells/well were cultured in 96-well plates in triplicate. The culture medium was replaced by medium supplemented with various concentrations of CA3 on the second day. Subsequently, the Cell Counting Kit-8 assay (CK04, Dojindo, Japan) was applied at 48 hours. The IC_50_ values were determined from the inhibition curves (log inhibitor concentration vs percentage of inhibition).

### Immunofluorescence

The cell lines were seeded on circular coverslips at 50% confluence and fixed with 4% paraformaldehyde following their respective treatments. After permeabilization using Triton X-100, samples were blocked for 1 hour at room temperature with PBS containing 5% bovine serum albumin. Following an overnight incubation at 4°C and after washing with PBS, Alexa Fluor® 488 anti-rabbit IgG (4340, Cell Signaling Technology, USA) was used as the secondary antibody. Nuclei were stained with DAPI (0100-20, Southern, USA). The images were observed and photographed using a positive fluorescence microscope.

### Animal studies

All experimental procedures were approved by the Animal Welfare Committee of the Ninth People's Hospital, Shanghai Jiao Tong University School of Medicine (Shanghai, China). Lentivirus transfected RSC96 (1.5×10^5^ cells per injection) was subcutaneously injected into a BABL/c null murine model. After a 2-week gavage with CA3 or selumetinib, according to the calculated IC_50_ values, the weights of the mice and the volumes of the tumors were measured. The exact volume of tumor was directly analyzed via conventional internal drainage method.

### Statistical analysis

The results are presented as the mean ± standard error of the mean. Differences between groups were assessed using one-way analysis of variance followed by Tukey's test. A *P*-value less than 0.05 (*P* < 0.05) was considered to indicate a significant difference. All analyses were conducted using SPSS (version 17.0) software (SPSS Inc., USA).

## Results

### Increased nuclear YAP is detected in pNF of the head and neck

**Figure 1:**
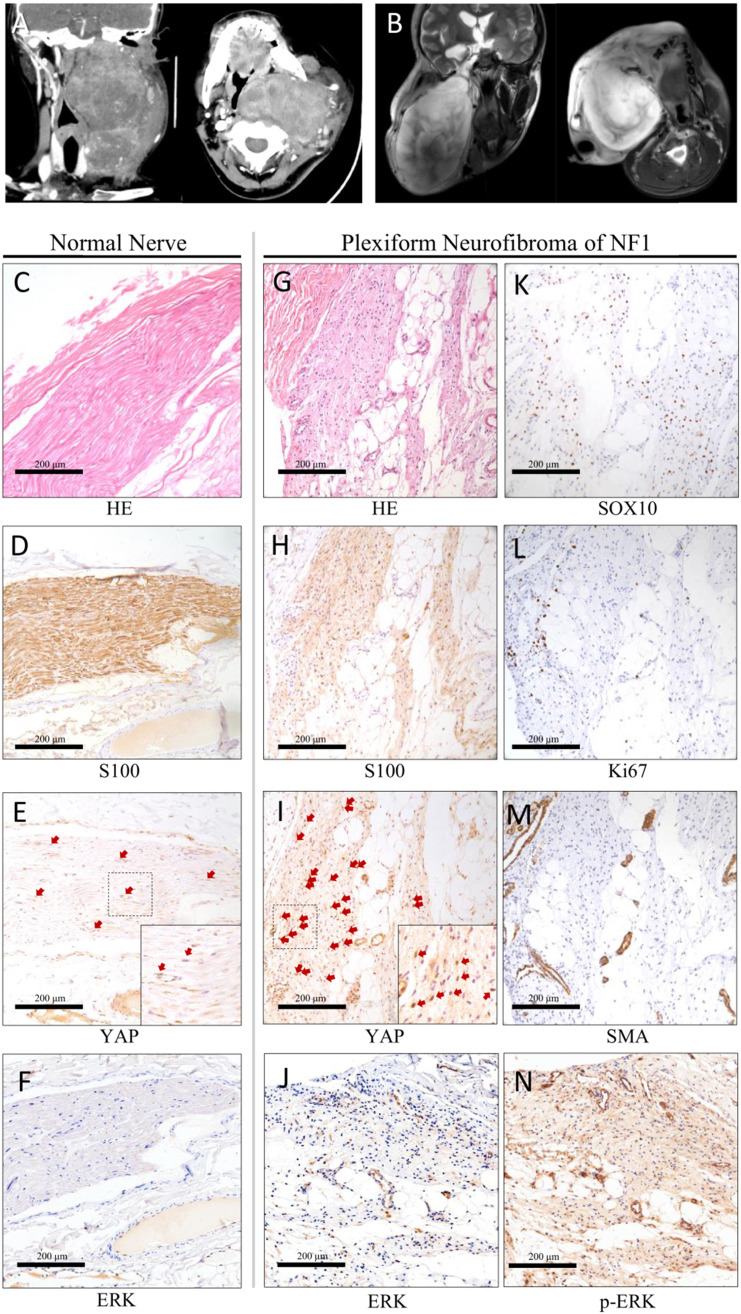
Typical clinic CT(**A**) and MRI (**B**) scan of the head and neck. Axial projection scan revealing the notably large lesion located in the maxillofacial-parapharyngeal region beneath the skull base. Coronal projection scan showing the narrowed pharyngeal cavity. Histopathological examinations of normal nerve tissue (**C**-**F**) showing confirmatory S100/ERK labeling and less nuclear yes-associated protein (YAP) translation of Schwann cells. Histopathological examinations of pNF (**G**-**N**). Diagnosis of the Schwann cell region in pNF was achieved with hematoxylin-eosin staining (**G**), positive reactions for S100 (**H**) and SOX10 (**K**), ERK (**J**) and p-ERK (**N**), and low identification of Ki67 (**L**) and SMA (**M**). Increased nuclear YAP translation of Schwann cells was identified (**I**). Bar = 200 μm.

In all, 19.2% of the diffuse pNF were located in the head and neck 15. A notably large and slow-growing neurofibroma in the head and neck could have serious effect on patient life, including dysfunction, pain, disfigurement, hemorrhage, and asphyxia. To confirm the expression pattern of YAP in pNF in the head and neck, we collected the records of 17 patients (aged 4-44 years) (Table 1). The ratio of males to females was 8:9 (0.89:1). All patients were diagnosed with pNF according to histopathological examinations, physical examination, and medical history. All patients suffered from café-au-lait macules and pNF. Five patients (5/17, 29.4%) with a clear family history were recorded. The typical radiography and histopathological examination of one classic patient are displayed (Figure 1). The notably large pNF in the head and neck could cause significant facial disfigurement and functional dysfunction (Figure 1A, B). Histologically, diagnosis of the Schwann cell region in the pNF was achieved with hematoxylin-eosin (Figure 1G), positive reactions for S100 (Figure 1H), SOX10 (Figure 1K), ERK (Figure 1J) and p-ERK (Figure 1N), and low identification of Ki67 (Figure 1L) and SMA (Figure 1M). According to this histological partition, compared with normal nerve tissue (Table 1, Figure 1C-G), the phenomenon of increased nucleus YAP was detected in the Schwann cell region of pNF (Table 1, Figure 1I). Nucleus YAP is the functional state. The average nuclear staining rate of YAP in the Schwann cell region was evaluated in all patients. The entire mean nuclear staining rate in the Schwann cell region was 67.11% (Table 1). We showed that the expression and nuclear distribution of YAP was persistently increased. No significant difference was observed for the average nuclear staining of YAP among patients with a family history or not (63.2% vs. 68.75%, *P* = 0.5082).

### Identification of an increased Yap in-nuclear process in *shNF1* Schwann cells

**Figure 2:**
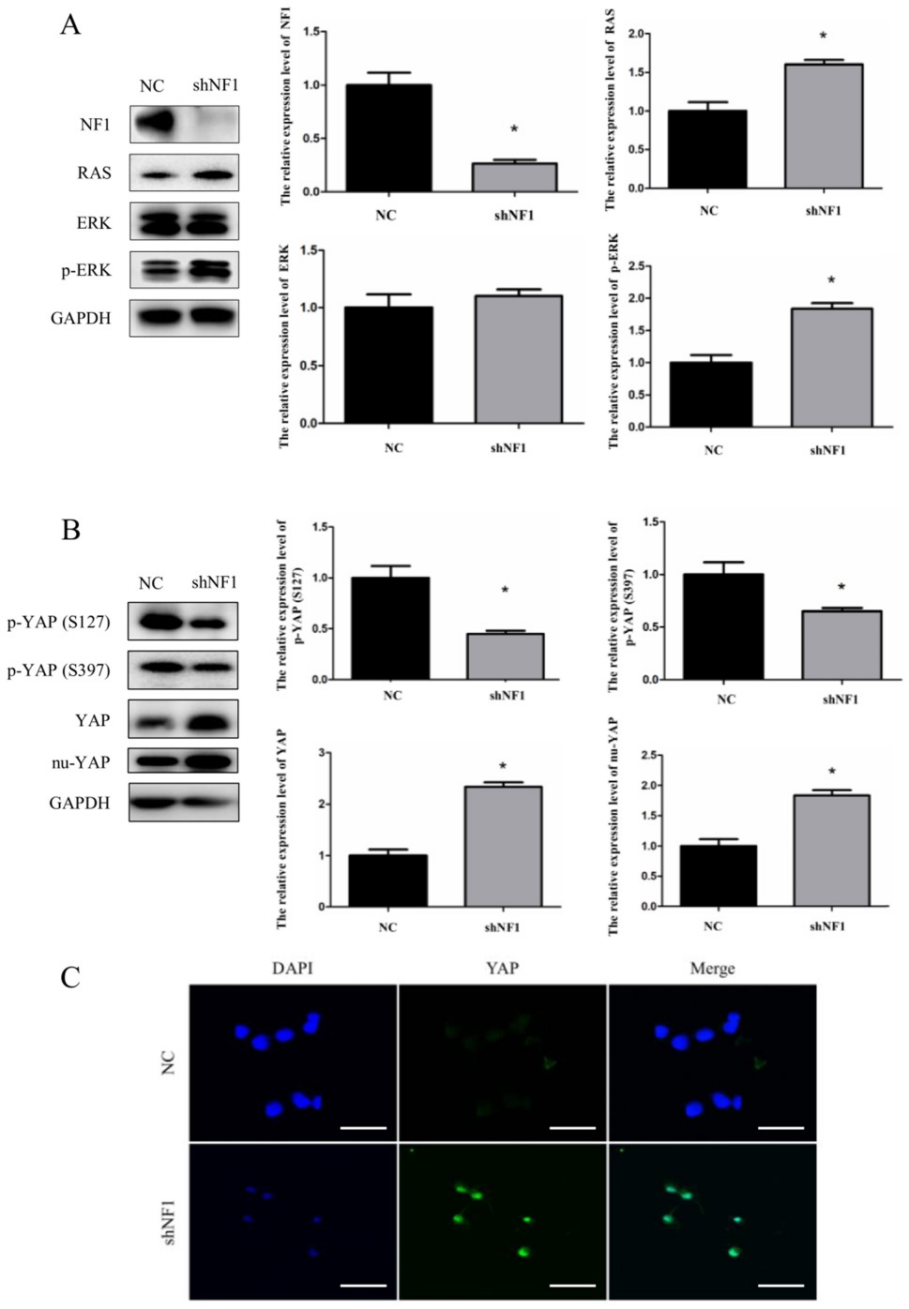
Downregulated phosphorylation and increased nuclear accumulation of yes-associated protein (YAP) in *shNF1* Schwann cells. (A) Immunoblot confirmation of NF1, RAS, ERK, p-ERK, and GAPDH in *shNF1* Schwann cells. (B) Immunoblots of p-YAP (S127), p-YAP (S397), YAP, nu-YAP, and GAPDH in *shNF1* Schwann cells. (C) Increased nuclear accumulation of YAP in *shNF1* Schwann cells. Bar = 20 μm. *P<0.05, based on Student *t*-test.

To further identify the YAP in-nuclear process under the *NF1^-/-^* status, we established the *shNF1* Schwann cell model (RSC96 cell line). To ensure the relevance and reliability of previously published research, we first examined the expression of NF1, RAS, ERK, and p-ERK in *shNF1* Schwann cells (Figure 2A). The expression of NF1 was blocked under the interference of *shNF1*, and consistent with the previous work, upregulated expression of RAS, ERK and p-ERK was also detected in *shNF1* Schwann cells (Figure 2A). Based on this reliable cellular model, analysis of the functional status of YAP was conducted. Considering that nuclear Yap is the functional state, phosphorylation of YAP indicates degradation and dysfunction status in the biological process. We observed that p-YAP (Ser127) and p-YAP (Ser397) were significantly decreased and the expression of total YAP and nuclear YAP was increased in the* shNF1* Schwann cell model (Figure 2B). Additionally, from the aspect of expression and functional state, we confirmed upregulated YAP in the *NF1^-/-^* background. According to a confocal immunofluorescence assay, from the point of view of subcellular localization, our results further confirmed that the interference of *shNF1* substantially promoted YAP nuclear translocation (Figure 2C).

### YAP inhibitor CA3 suppresses cellular proliferation

**Figure 3:**
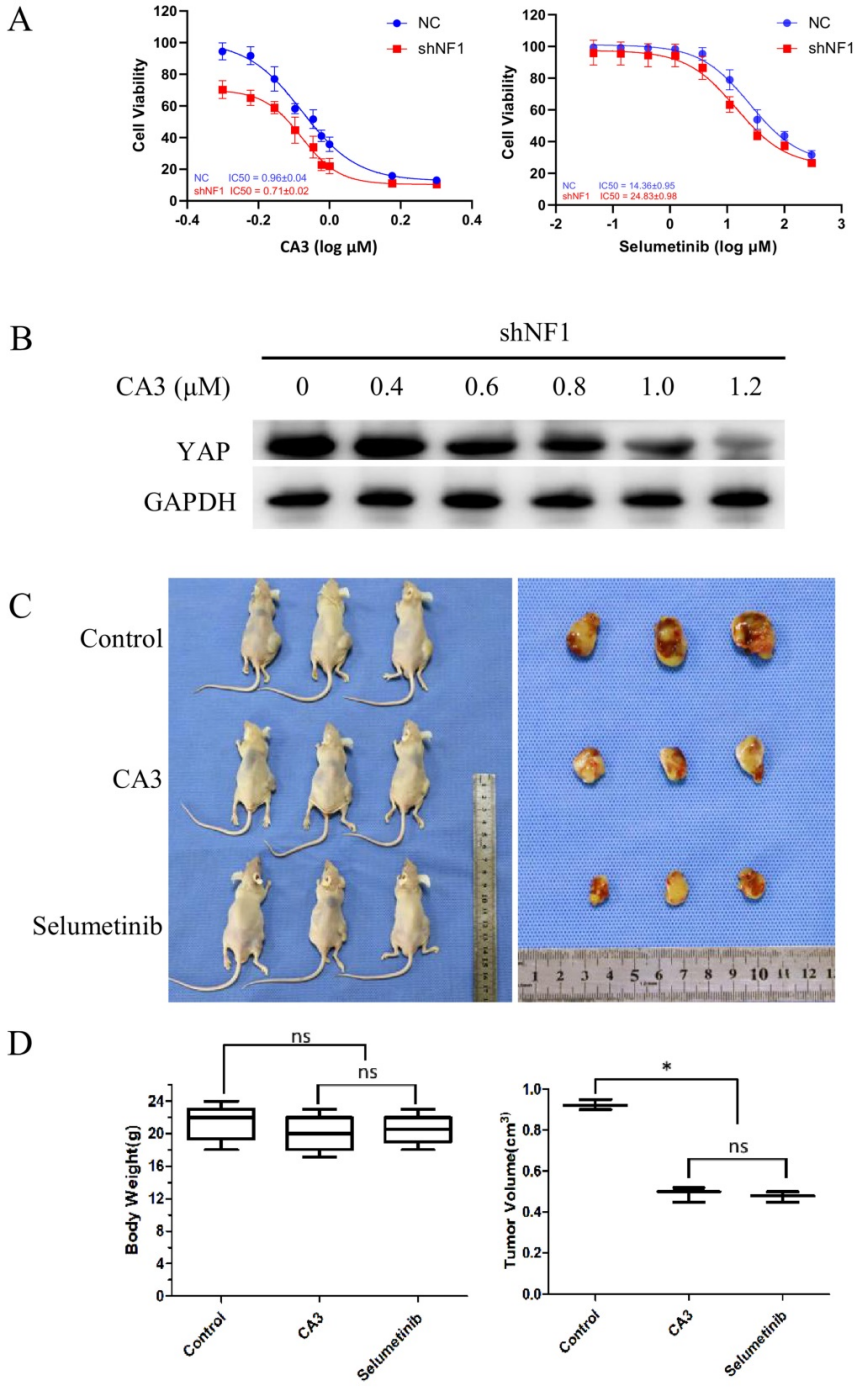
*NF1* knockout sensitizes Schwann cells to the yes-associated protein (YAP) inhibitor CA3. (A) *shNF1* Schwann cells were treated with increasing concentrations of CA3 and selumetinib. Cellular viability was determined by the CellTiter-Glo assay, and the IC50 values were calculated based on a nonlinear regression analysis. (B) Immunoblot confirmation of downregulated YAP in *shNF1* Schwann cells treated with increasing concentrations of CA3. (C) Treatment with CA3 or Selumetinib reduces the tumor size in a mouse model generated by subcutaneous injection of *shNF1* Schwann cells. (D) Quantification of body weight among the different treatment groups. (E) Quantification of tumor volume among the different treatment groups at 15 days after tumor implantation. *P<0.05, based on Student's *t*-test. ns, no significant difference.

To investigate the potential role of YAP in regulating Schwann cell survival, we examined the effects of inhibiting YAP with the pharmacological inhibitor CA3. As discussed previously, selumetinib, the classic selective inhibitor for MEK1/2, was selected as another matched group. As shown, CA3 and selumetinib can induce a concentration-dependent decrease in cell viability in *shNF1* Schwann cells (Figure 3A). Lentivirus transfected RSC96 (1.5×10^5^ cells per injection) was subcutaneously injected into a BABL/c null murine model.5). To examine the defined blocking effect of CA3 on the expression of YAP, we further analyzed the expression of YAP protein in *shNF1* Schwann cells. Downregulated YAP was detected under treatment with increasing concentrations of CA3 (Figure 3B). We also explored the effect of YAP *in vivo* using immunocompromised mice (Figure 3C and D). The results showed that YAP knockdown significantly inhibited tumor growth. For the inhibition effect, no significant difference was observed between the CA3 group and the selumetinib group. Blockage of the expression of YAP and CA3 might exhibit a similar but more sensitive effect on *shNF1* Schwann cells. For the toxicity responses, no obvious difference in body weight was noted among different groups.

## Discussion

With respect to the potential transformation from pNF to malignant peripheral nerve sheath tumors (MPNSTs), differential diagnosis of MPNSTs and other neurogenic tumors could be a basic but vital step. Except for clinical signs or symptoms according to the diagnostic criteria approved by the National Institutes of Health, histological diagnosis is still the most important evidence. The key features of various NF1-associated nerve sheath tumors are summarized according to the classification proposed by the National Institute of Health (NIH) in 2016. The immunohistochemistry of neurofibroma or pNF includes extensive but not diffuse S100 positivity in the Schwannian component and SOX10 positivity; a lattice-like CD34+ fibroblastic network; a less than 3% Ki67 labeling index; diffusely enlarged nerves that need to be replaced, often involving multiple nerve fascicles; and EMA+ perineurial cells 16. Thus, all patients in our study were diagnosed with pNF according to histopathological examination, physical examination, and medical history. All patients suffered from café-au-lait macules and pNF. According to medical records, only five patients (5/17, 29.4%) were recorded with a clear family history. Regardless of family history, all NF1 patients suffered from café-au-lait macules, and pNF might carry diverse *NF1* mutations 17.

According to our research on the histological analysis and cellular model, increased nuclear translation of YAP was confirmed under the *NF1^-/-^* background. The Hippo pathway is a highly conserved pathway in different species. The core of the Hippo pathway in mammals consists of a kinase cascade; MST1/2 and LATS1/2; downstream effectors; transcriptional coactivators, YAP and TAZ; and TEAD family transcription factors 13. Commonly, cytoplasmic phosphorylation of YAP/TAZ by upstream casein kinase 1 leads to its ubiquitination and proteasomal degradation 18. Thus, dephosphorylation and nuclear translation of YAP is the functional state. Activated TAZ/YAP-TEAD can directly induce the oncogenic process, including platelet-derived growth factor receptor (PDGFR) signaling and other signals. Thus, Schwann cells overexpressing YAP^S112A^ (YAP phospho-mutant) can display a precursor/stem-like and highly proliferative state 18. Additionally, YAP/TAZ is highly required for Schwann cells to remyelinate axons 19. According to observation of the *NF1*-related mouse model, nonmyelinating Schwann cells have also been identified 20, 21, which implies the vital role of remyelination related to *NF1*. For the direct regulation relationship between YAP and NF1, the Hippo-YAP pathway has been demonstrated to be regulated by pyruvate dehydrogenase kinase-1 (PDK-1) 22, which is involved in the RAS-PI3K-PDK regulation axis 23. However, no clear evidence exists declaiming this issue.

Compared with a previous descriptive study on nuclear YAP of pNF 11 and based on more reliable histological verification and calculation, we further explored a promising application for the treatment target. Among diverse inhibitors applied in clinical trials related to pNF, the selective inhibitor for MEK1/2 selumetinib (AZD6244, ARRY-142886) is the only treatment available for children with symptomatic pNF approved by the FDA 6. Compelled by the efficiency of selumetinib, selumetinib and other MEK inhibitors have also been gradually included in clinical trials for adolescents or adults with inoperable pNF 24. With application in additional clinical trials, the deficiencies and side reactions of selumetinib have also received additional attention. Most controversial discussions of selumetinib are on its notably slow shrinkage of pNF, its long-term course of treatment, the duration of treatment required to sustain a clinical benefit, and whether the patient benefits from this medicine 6, 7, 9. According to research related to cancers, different potential factors might play various roles in the acquired resistance and efficiency of selumetinib, such as KRAS/BRAF mutation status, abnormal ERK activity, hyperactivation of the PI3K-mTOR pathway, and upregulated scaffold protein CEMIP via a Wnt-dependent pathway 25-27. Thus, attempts to explore novel or adjuvant targets are still needed to develop or repurpose compounds for the treatment of pNF. Combined with the background, YAP was reported to mediate resistance to MEK inhibition in neuroblastoma with hyperactivated RAS signaling through transcriptional activation of E2F and MYC 10. According to the increased functional state of YAP in pNF, we compared the YAP inhibitor CA3 and selumetinib in this research. Compared with control Schwann cells, according to the IC_50_ value, CA3 might exert a more sensitive effect on *shNF1* Schwann cells than selumetinib. However, for the inhibition effect in the *in vivo* assays, no significant difference was observed between the CA3 group and the selumetinib group. In the future, a larger sample size and screening of the drug concentration gradient might supply additional details on the difference between YAP inhibitor and selumetinib. This study has important clinical implications because potential combinatorial inhibition of MEK1/2 and YAP signaling might be an effective combination to circumvent the reprogramming and nonmyelination of Schwann cells. Although no Hippo pathway modulating drugs have currently been tested in the clinic, increasing interest has appeared within academia and industry in the development of inhibitors of YAP activity 28.

## Conclusion

In summary, we demonstrated that increased nucleus YAP was histopathologically detected in pNF of the head and neck. Based on the *shNF1* Schwann cell model, increased nuclear translation and the functional state of YAP imply that the Hippo pathway might play an important role in the formation of pNF. Compared with a selective inhibitor for MEK1/2, the YAP inhibitor could exert a similar but more sensitive effect on *shNF1* Schwann cells. All of these observations support YAP as a novel or adjuvant therapy target in treatment of pNF.

## Figures and Tables

**Table 1:** Increased nuclear YAP staining detected in pNF of the head and neck.

ID	Age	Gender	Family history	Café-au-lait macules	L/R	Location	S100	YAP average staining (nuclear, %)
Normal nerve								
	25	M	N	N	L	Nervous cutaneous femoris lateralis	+	13
pNF								
1	4	F	N	Y	L	Neck	+	56
2	24	F	N	Y	R	Face	+	75
3	44	F	N	Y	R	Face	+	80
4	15	M	N	Y	R	Face	+	35
5	22	F	N	Y	L	Nasal and submandibular	+	84
6	29	F	N	Y	R	Face	+	86
7	19	F	Y	Y	R	Face	+	78
8	22	F	Y	Y	R	Neck	+	69
9	24	M	Y	Y	R	Face and neck	+	62
10	18	M	N	Y	R	Face	+	42
11	17	M	Y	Y	R	Tempus	+	63
12	14	M	N	Y	R	Face and neck	+	76
13	18	M	N	Y	L	Face	+	71
14	7	M	N	Y	L	Face	+	71
15	14	M	N	Y	R	Face	+	81
16	26	F	N	Y	R	Lip and neck	+	68
17	14	F	Y	Y	L	Neck	+	44

Abbreviations: M, Male; F, Female; N, No; Y, Yes; L, Left; R, Right.
